# Establishment of Integrated Quality Regions for the Rare Medicine Food Homology Plant *Cyclocarya paliurus* (Batal.) Iljinsk in China

**DOI:** 10.3390/biology14121639

**Published:** 2025-11-21

**Authors:** Heng Jiang, Haijun Chen, Haiming Wang, Bin Huang, Ting Chen

**Affiliations:** 1 Sino-Pakistan International Center on Traditional Chinese Medicine, School of Pharmaceutical Sciences, Hunan University of Medicine, Huaihua 418000, China; JH15874556538@163.com (H.J.); chenhj@hnmu.edu.cn (H.C.); 2 Key Laboratory of State Administration of Traditional Chinese Medicine for Production & Development of Cantonese Medicinal Materials, Guangzhou Comprehensive Experimental Station of National Industrial Technology System for Chinese Materia Medica, Guangdong Engineering Research Center of Good Agricultural Practice & Comprehensive Development for Cantonese Medicinal Materials, School of Chinese Materia Medica, Guangdong Pharmaceutical University, Guangzhou 510006, China; 3 The College of Traditional Chinese Medicine, Yunnan University of Chinese Medicine, Kunming 650500, China; whmvcd@163.com

**Keywords:** MaxEnt, ArcGIS, *Cyclocarya paliurus* (Batal.) Iljinsk, medicine food homology plant, integrated quality regions

## Abstract

This study focuses on the plant *Cyclocarya paliurus* (Batal.) Iljinsk, which is unique to China and is characterized by its dual role as both a medicine food homology plant. We developed a novel framework termed “integrated quality regions”, which combines ecological suitability with the spatial distribution of bioactive compounds. Our findings indicate that the distribution of *C. paliurus* is primarily regulated by precipitation and temperature factors. Currently, the most suitable habitats are concentrated in the provinces of Jiangxi, Zhejiang, and Hunan; however, under future climate scenarios, the centroid of suitable habitats is projected to shift northwestward. The accumulation of key bioactive compounds, specifically quercetin and kaempferol, is significantly correlated with the precipitation of warmest quarter and the aspect. Through spatial integration analysis, we identified the border region between Guangdong, Hunan, and Guangxi (centering on Yizhang County, Linwu County, and Renhua County) as a potential area for integrated quality regions, characterized by both ecological suitability and high medicinal value. Despite limitations in the sample size, the ecological–chemical synergistic zoning model proposed in this study provides a scientific basis for the conservation, optimal cultivation site selection, and sustainable utilization of *C. paliurus* resources.

## Simple Summary

This study developed an integrated quality assessment system for the rare medicinal plant *Cyclocarya paliurus* (Batal.) Iljinsk. in China. By combining species distribution data, environmental variables, climate projections, and measured compound content (quercetin and kaempferol), a predictive model was created. The results show that suitable habitats, currently concentrated in provinces like Jiangxi and Hunan, are projected to shift northwestward under future climate scenarios. The content of key compounds was significantly linked to specific environmental factors. The research identifies the border region of Guangdong, Hunan, and Guangxi provinces as the optimal integrated quality area. This work provides a multi-scale decision-making framework for the conservation and sustainable use of this valuable plant.

## 1. Introduction

The medicinal properties of plants primarily arise from secondary metabolites such as alkaloids, flavonoids, and terpenoids [1], in stark contrast to crops that are centered around primary metabolites like carbohydrates and proteins. The synthesis and accumulation of these secondary metabolites are significantly influenced by environmental factors associated with their geographical distribution [2]. Key factors that determine the geographical distribution of medicinal plants include climatic factors (e.g., annual average temperature and precipitation), soil characteristics (e.g., pH and nutrient status), and topographical features (e.g., elevation and slope). Together, these factors form the foundation of species distribution models [3,4,5]. The geographical distribution of these plants interacts with their physiology through the aforementioned environmental factors, where environmental stressors (such as low temperatures and salinity) play a crucial role. These stressors often act as signals that activate specific secondary metabolic pathways in plants, leading to the accumulation of pharmacologically active compounds, such as flavonoids, terpenes, and phenolics [6,7]. For instance, the accumulation of anthraquinones and flavonoids in *Rheum tanguticum* has been found to correlate closely with environmental factors such as annual precipitation, annual average temperature, and soil pH [8]. This stress-induced metabolic reconfiguration suggests that the ecological optimum for biomass accumulation in medicinal plants may spatially differ from the optimum for the accumulation of quality pharmacologically active components. In some species, such as *Atractylodes lancea*, the regions with the highest quality medicinal materials are located at the margins of their geographical distribution [9], demonstrating the critical role of moderate stress in driving the formation of pharmacologically active components.

Given the potential spatial disjunction between ecological suitability and quality suitability, the scientific planning of traditional Chinese medicine production areas must adopt differentiated strategies. For species where ecological and quality suitability highly overlap, traditional ecological suitability zoning methods (such as integrating GIS with the MaxEnt model) can be employed [10,11]. However, in the more common scenario of spatial heterogeneity, it is necessary to construct a multidimensional evaluation system that integrates ecological factors with active ingredient analysis [12,13]. This includes the application of models such as geographically weighted regression to reveal the spatial heterogeneity of environmental-component relationships [14,15]. Ultimately, a weighted overlay of ecological suitability and quality suitability layers should be conducted to create production zoning that balances both yield and quality [16]. This strategy transcends the traditional model that focuses solely on yield, aiming to achieve a unified approach to the authenticity of medicinal materials and the sustainable utilization of resources.

*Cyclocarya paliurus* (Batal.) Iljinsk., is a renowned medicinal and edible plant in China [17]. Its leaves are rich in polysaccharides and flavonoids [18], which are secondary metabolites known for their significant hypoglycemic and lipid-regulating activities [17]. Consequently, it has garnered widespread attention in the fields of functional foods and traditional Chinese medicine [17,19]. However, due to overharvesting and habitat destruction, its wild resources have been classified as endangered [20], making the need for sustainable utilization urgent. Current resource assessment studies on *C*. *paliurus* primarily focus on ecological suitability (i.e., the locations where the species can grow) using models such as MaxEnt to predict its potential distribution [21,22]. Nonetheless, these studies often overlook quality suitability (i.e., the locations where the species can effectively accumulate bioactive compounds) and fail to consider the regulatory effects of environmental factors on the synthesis of secondary metabolites. This oversight has resulted in challenges in accurately identifying cultivation areas that possess both high yield and high-quality potential, representing a critical gap in current research.

To address these limitations, this study proposes a spatial evaluation framework that integrates ecological and quality suitability. We hypothesize that high-quality regions for *C*. *paliurus* are not confined to its ecologically optimal areas but are instead distributed in environments that can stimulate specific secondary metabolic pathways. To test this hypothesis, we will combine the MaxEnt model with spatial interpolation analysis of key bioactive compounds, aiming to: (1) identify the dominant environmental factors influencing the distribution and quality formation of *C*. *paliurus*; (2) delineate its integrated quality regions; and (3) propose strategies for its sustainable utilization and conservation. This approach transcends the traditional focus on species survival, providing a more scientifically informed basis for the quality production and effective conservation of *C*. *paliurus* resources.

## 2. Materials and Methods

### 2.1. Species Data Acquisition and Processing

**Figure 1:**
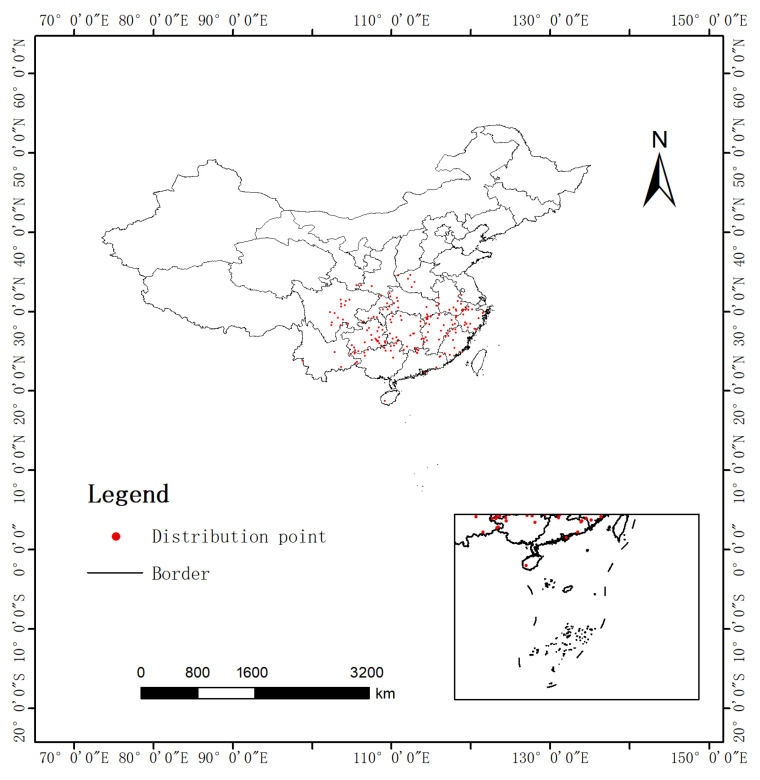
The distribution information of *C. paliurus*.

Geographical distribution data for *Cyclocarya paliurus* (Batal.) Iljinsk. were compiled from three databases: the Chinese Virtual Herbarium (CVH, http://www.cvh.ac.cn, accessed on 10 March 2024), the National Specimen Information Infrastructure (NSII, http://www.nsii.org.cn/2017/home.php, accessed on 12 March 2024), and the Global Biodiversity Information Facility (GBIF, https://www.gbif.org/, accessed on 15 March 2024). Latitude and longitude coordinates for all samples were organized into a CSV file and imported into ArcGIS (version 10.8; ESRI, Redlands, CA, USA). To minimize spatial autocorrelation, the “Spatially Rarefy Occurrence Data for SDMs” function within the “SDM Toolbox” was employed to filter out samples located less than 10 km apart [23]. As illustrated in Figure 1, a total of 170 sample points were retained for subsequent modeling. Flavonoids, specifically quercetin and kaempferol, are the primary active compounds found in the leaves of *C. paliurus*, characterized by their abundant presence and strong association with health-promoting effects. Given that this species is not yet included in the “Chinese Pharmacopoeia”, this study establishes quercetin and kaempferol as key quality control indicators based on the quality control requirements outlined by the Jiangxi Provincial Drug Administration’s traditional Chinese medicine standards (JXYCBZ2023-002) [24]. A systematic literature search was conducted using the China National Knowledge Infrastructure (CNKI) (https://www.cnki.net, accessed on 15 May 2024) and Google Scholar (https://scholar.google.com, accessed on 15 May 2024), focusing on publications from 2008 to 2020 related to sampling analysis and flavonoid component detection in *C. paliurus*. After removing duplicates and verifying data integrity, a comprehensive dataset comprising 26 records of quantitative measurements of quercetin and kaempferol was compiled (Supplementary Table S1). It is important to note that the sample size used for chemical composition analysis (n = 26) is relatively limited, which may introduce uncertainty in predictions at a national scale. However, these samples cover the main distribution provinces of *C. paliurus*, capturing the ecological gradients of its core distribution area as comprehensively as possible. All chemical composition data are sourced from the literature utilizing standardized quantitative methods, such as high-performance liquid chromatography, to ensure data comparability.

### 2.2. Environmental Variables Optimization

The environmental variables associated with the habitat of *C. paliurus* selected for this study include bioclimatic variables, soil factors, and topographic factors. Bioclimatic data were obtained from the WorldClim database (http://www.worldclim.org/; accessed on 28 April 2022) at a resolution of 2.5 arc minutes, which includes 19 bioclimatic variables for both baseline (“current”) (1970–2000) and future climate scenarios. The BCC-CSM2-MR model, recognized for its robust performance in simulating climate in China, was utilized to project future climate changes [25]. Two Shared Socioeconomic Pathways (SSPs) were selected from the BCC-CSM2-MR model: a low carbon emission scenario (SSP126) and a high carbon emission scenario (SSP585). SSPs are commonly employed for forecasting global social and climate scenarios, and they effectively account for uncertainties in future projections [26]. SSP585 signifies a high-forcing scenario characterized by a peak population, low technological innovation, slow energy improvements, and sustained high energy demand, whereas SSP126 represents a low-forcing scenario [27]. Each SSP scenario was further categorized into two distinct periods: the 2050s (2041–2060) and the 2090s (2081–2100) [28]. Soil and topographic variables were sourced from the Harmonized World Soil Database (https://www.fao.org/soils-portal/data-hub/soil-maps-and-databases/harmonized-world-soil-database-v12/en/; accessed on 15 October 2022) and the WorldClim database (http://www.worldclim.org/; accessed on 18 October 2022). All aforementioned environmental variables (Table 1) were originally global data, which were subsequently clipped to the borders of China using ArcGIS (version 10.8). To construct a robust and interpretable species distribution model, environmental variables were selected using a two-step approach to eliminate multicollinearity. First, based on the variable contribution rates from an initial MaxEnt (version 3.4.4), variables that did not contribute to species distribution predictions were removed. Second, Pearson correlation analysis and variance inflation factor (VIF) calculations were performed on the remaining variables. If any variables were found to be highly correlated (|r| > 0.8), the variable with the higher contribution rate and clear ecological significance was retained [29] until all remaining variables had VIF values below 10 [30]. This method preserves effective information while ensuring that the final set of variables maintains both independence and ecological relevance, thereby enhancing the reliability and interpretability of the model.

### 2.3. Predictive Modeling and Data Analysis

Species distribution models were constructed using MaxEnt (version 3.4.4). The model parameters were set as follows: 75% of the distribution data were used for training, while 25% were used for testing; the regularization multiplier was set to 1; the maximum number of background points was 10,000; the maximum number of iterations was 1,000,000, with a convergence threshold of 0.00001. To assess model stability, 10-fold cross-validation was performed, and the option to “remove duplicate records” was enabled. In the feature combination optimization, various settings including L, LQ, H, LQH, and LQHP were tested, ultimately selecting the LQH combination (linear, quadratic, and hinge features) with a regularization multiplier of 1 to balance model complexity and generalization capability. Model performance was evaluated using the average area under the receiver operating characteristic curve (AUC) from the 10-fold cross-validation, with the output format being Cloglog, indicating the probability of presence under given environmental conditions. The contribution and significance of environmental factors were evaluated using a Jackknife test, and the optimal ranges indicated by the response curves of these factors were identified [31]. The ASCII files generated from the MaxEnt prediction analysis represented the probability of *C. paliurus* occurrence, denoted as *p*, with values ranging from 0 to 1. A threshold of *p* ≥ 0.5 was established to determine the optimal suitable range of important environmental factors, with the peak probability of occurrence serving as the adaptive threshold for these factors [28]. The ASCII files were subsequently imported into ArcGIS (version 10.8) and converted into raster files. The Reclassify function in the raster calculator of the Spatial Analyst Tools was utilized to merge the predictions. The resulting suitability index was categorized into four classes using Jenks’ natural breaks method: non-suitable habitat (0~0.1), low-suitable habitat (0.1~0.3), moderately suitable habitat (0.3~0.5), and highly suitable habitat (0.5~1) [32]. Finally, the attribute table of the reclassified files was employed to calculate the ratio of the grid numbers for each class to the total number of grids, thereby estimating the potential habitat area for *C. paliurus*.

### 2.4. Centroid Migration

The centroid of the suitable habitat for *C. paliurus* under various scenarios and periods was determined using the “Mean Center” function within the spatial statistics module of ArcGIS (version 10.8). This analysis elucidated the position of the centroid and the alterations in the suitable habitat of *C. paliurus* in response to different climate change conditions, thereby facilitating further examination of centroid migration trends [33].

### 2.5. Integrated Quality Regions

A Spearman correlation analysis, which does not require data to be normally distributed, was conducted to assess the relationship between quercetin and kaempferol concentrations and 37 environmental variables across 26 samples of *C. paliurus*. This analysis aimed to identify key environmental factors that significantly influence flavonoid accumulation. Based on the identified significant variables, a spatial distribution model of the compound concentrations was constructed using the co-kriging method within the statistical analysis module of ArcGIS 10.8 [34]. Model parameters were automatically optimized using an empirical semi-variogram function and the interpolation accuracy was evaluated through leave-one-out cross-validation.

To integrate ecological and quality spatial information, ecological suitability rasters (derived from MaxEnt model outputs with original values ranging from 0 to 1) were combined with quality suitability rasters (obtained from co-kriging interpolation results and normalized to a 0~1 range based on quercetin and kaempferol concentrations). Both rasters were standardized to create a continuous suitability index. A weighted linear combination method was employed, assigning equal weights (0.5) to ecological and quality suitability, to perform spatial overlay and generate a comprehensive quality index that balances species survival potential with medicinal value. Finally, the comprehensive quality index was classified using the natural breaks method, resulting in a delineation map of the integrated quality regions for *C. paliurus*.

## 3. Results

### 3.1. Modeling Environmental Variables

**Figure 2:**
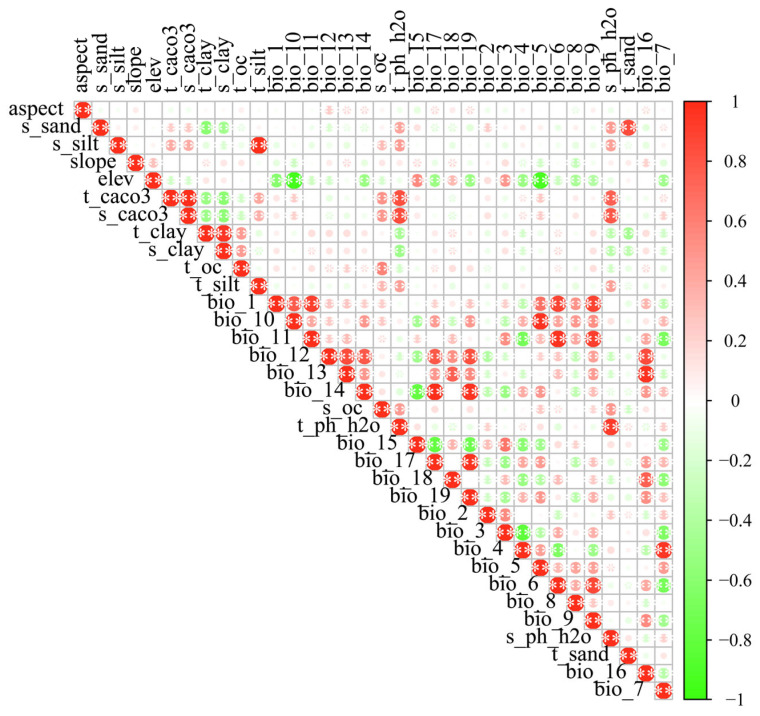
Environment variables correlation heatmap. This figure displays the Pearson correlation coefficients between pairs of environmental variables used for species distribution modeling. The color intensity represents the strength of the correlation, with red indicating positive correlations and green indicating negative correlations. Darker shades correspond to stronger correlations. “*” indicates a *p*-value ≤ 0.05, “**” indicates a *p*-value ≤ 0.01.

A Pearson correlation analysis was performed on the 34 environmental variables (Figure 2). Ultimately, 17 environmental factors were selected from the initial set of variables to construct the MaxEnt model (Table 2).

### 3.2. Model Accuracy Evaluation

**Figure 3:**
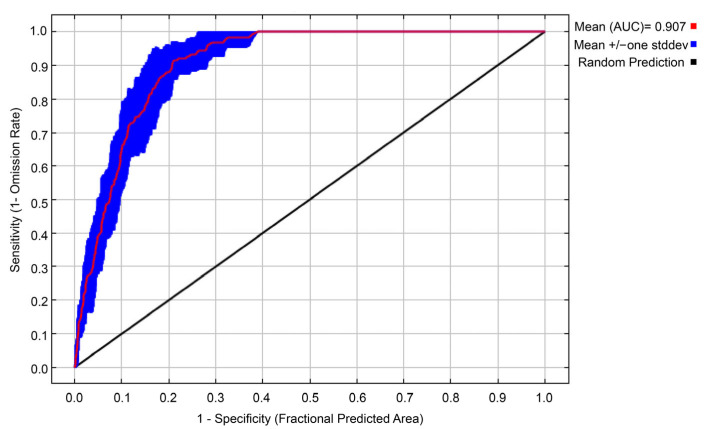
MaxEnt ROC curve.

The MaxEnt model demonstrated excellent predictive performance. The average AUC obtained from ten repetitions of cross-validation was 0.907, significantly higher than the baseline level of random prediction (AUC = 0.5) (Figure 3). This result confirms the model’s high reliability in identifying the potential distribution of *C. paliurus*.

### 3.3. Dominant Environmental Variables

**Figure 4:**
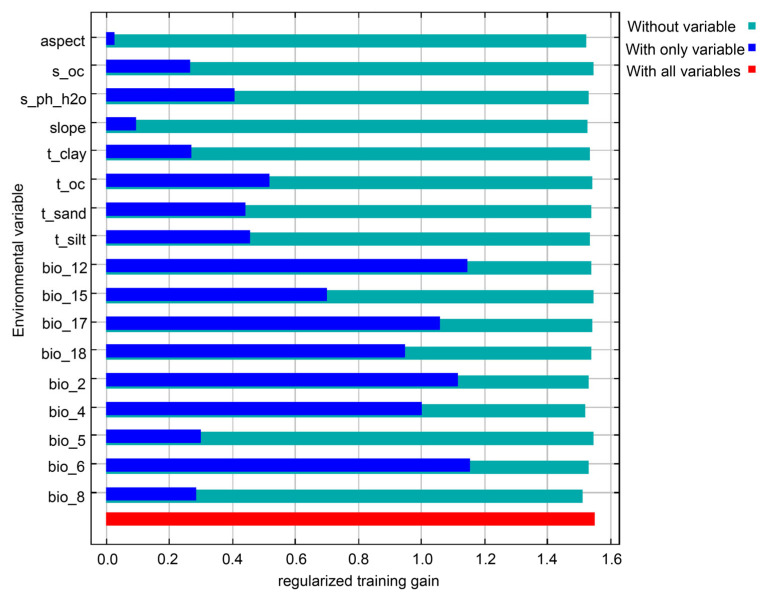
Variable gain. The jackknife test was performed to assess the relative importance of environmental variables in predicting the distribution of *C. paliurus*. The blue bars indicate the regularized training gain when each variable is used in isolation. The green bars show the training gain when the variable is omitted from the model. The red bar represents the training gain achieved using all environmental variables combined.

Based on a comprehensive analysis of contribution rates and regularization training gains, the dominant environmental factors influencing the distribution of *C. paliurus* have been identified. Under current climatic conditions, the Precipitation of the Driest Quarter (Bio17), Annual Precipitation (Bio12), Minimum Temperature of the Coldest Month (Bio6), Temperature Seasonality (Bio4), and Mean Diurnal Range (Bio2) collectively account for 84.1% of the model’s variance, with Bio17 contributing the most (34.0%) (Table 3). Jackknife tests further confirmed that these variables have significant independent effects, as indicated by their regularization training gains, all of which exceed 1 (Figure 4). The results suggest that the distribution of *C. paliurus* is primarily driven by the availability of precipitation (represented by Bio17 and Bio12), while extreme temperature (Bio6) and temperature variability (Bio4, Bio2) also serve as critical constraints, reflecting the species’ ecological dependence on the interplay of water and thermal conditions.

### 3.4. Suitable Habitat Range

**Figure 5:**
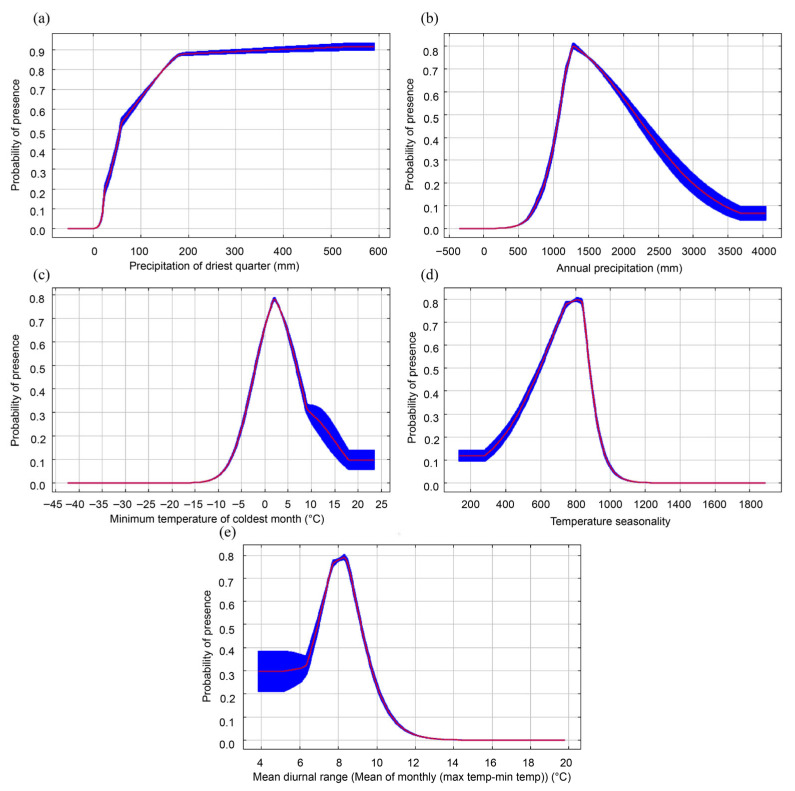
Dominant variables’ response curve. (**a**) Bio17 Precipitation of driest quarter; (**b**) Bio12 Annual precipitation; (**c**) Bio6 Minimum temperature of coldest month; (**d**) Bio4 Temperature seasonality; (**e**) Bio2 Mean diurnal range (Mean of monthly (max temp-min temp)).

Based on the response curves generated by the MaxEnt model (Figure 5), we identified the suitable ranges and ecological thresholds of five dominant environmental factors influencing the distribution of *C. paliurus*. The analysis revealed a pronounced nonlinear relationship between the species’ probability of survival and the various environmental factors. Specifically, the highest probability of occurrence for Precipitation of the Driest Quarter (Bio17) was observed at 563.2 mm, while the suitable range for Annual Precipitation (Bio12) was found to be between 1078.4 and 2172.6 mm. Additionally, the lower threshold for Minimum Temperature of the Coldest Month (Bio6) was determined to be −1.9 °C (Table 4). These quantitative results not only highlight *C. paliurus*’s specific dependence on hydrothermal conditions, but also reflect the primary limiting mechanisms of its distribution—namely, the combined effects of winter drought stress and low-temperature tolerance. Notably, the narrow suitable ranges for Temperature Seasonality (Bio4) and Mean Diurnal Range (Bio2) further indicate that the species has limited adaptability to temperature fluctuations. These findings elucidate the mechanistic basis for the distribution patterns of *C. paliurus* and provide critical ecological insights for the precise delineation of conservation areas.

### 3.5. Potential Distribution: Current Climate

**Figure 6:**
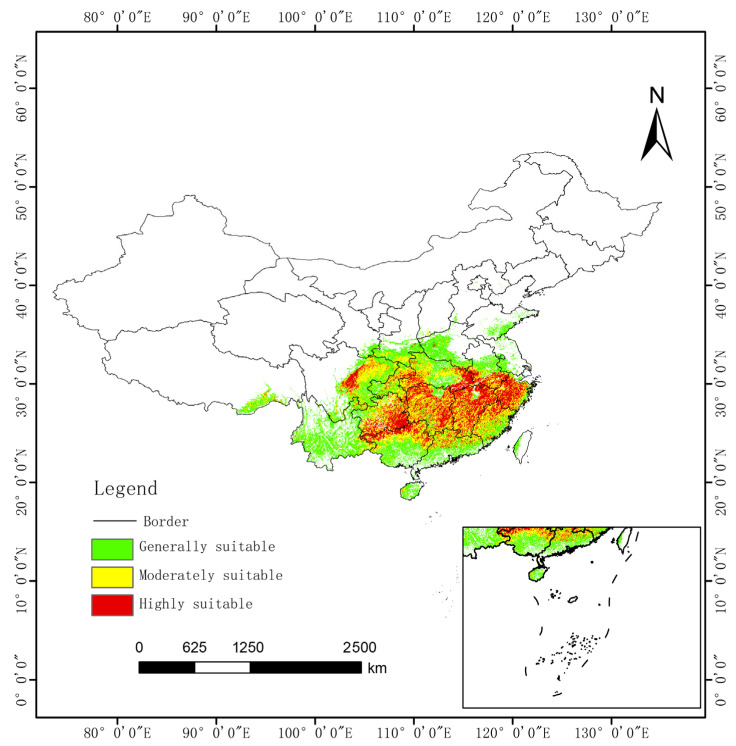
*C. paliurus* potential distribution (current climate).

Under current climate conditions, the potential suitable habitats for *C. paliurus* exhibit distinct latitudinal gradients and spatial aggregation characteristics (Figure 6). Highly suitable areas (covering an area of 45.95 × 10^4^ km^2^) are predominantly located south of the Yangtze River. This pattern is primarily regulated by the hydrometeorological conditions of the East Asian monsoon region: the highly suitable areas closely align with regions receiving annual precipitation greater than 1200 mm and where the minimum temperature in the coldest month exceeds −1.9 °C. This distribution pattern corroborates the ecological characteristics of *C. paliurus* as a subtropical tree species, indicating that its distribution is constrained by both winter low temperatures and seasonal drought. The fragmentation of suitable habitats around the Yunnan–Guizhou Plateau and the Sichuan Basin further highlights the critical role of topography in the redistribution of hydrometeorological resources.

### 3.6. Potential Distribution: Future Climate

**Figure 7:**
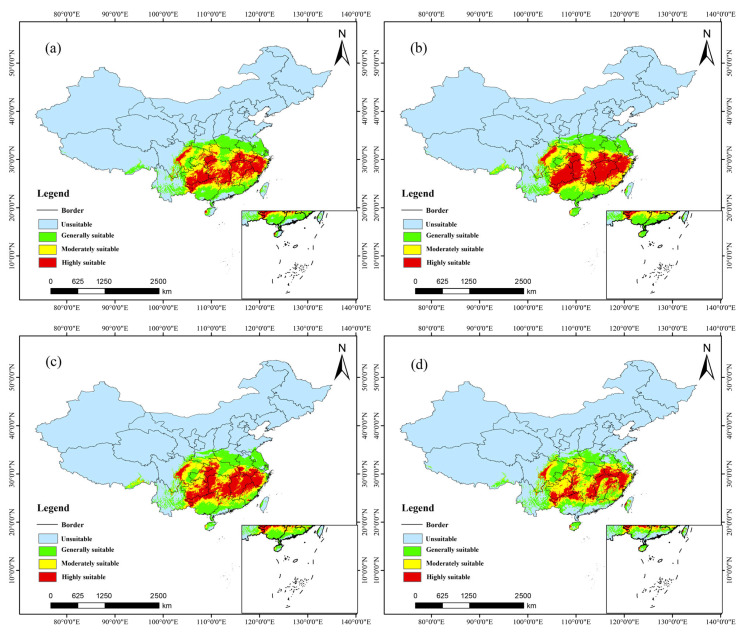
*C. paliurus* potential distribution (future climate): (**a**) 2041–2060, SSP126; (**b**) 2081–2100, SSP126; (**c**) 2041–2060, SSP585; (**d**) 2081–2100, SSP585.

Under current and future climate scenarios, the suitable habitat patterns for *C. paliurus* exhibit significant spatiotemporal dynamics, reflecting the profound impacts of different greenhouse gas emission pathways on its distribution (Figure 7, Table 5). In a low-carbon emission scenario (SSP126), the suitable habitat for *C. paliurus* shows a trend of continuous expansion. By the near term (2041–2060), the area of highly suitable habitat is projected to increase to 61.21 × 10^4^ km^2^, primarily located in the middle and lower reaches of the Yangtze River, the southwest region, and the southeastern coastal areas. By the end of the century (2081–2100), this area is expected to further expand to 74.57 × 10^4^ km^2^, with particularly notable growth along the coastal regions of Fujian. The total suitable area is projected to rise from the current 19.09% to 23.24%, indicating that under moderate climate change, *C. paliurus* may experience a broader potential distribution range.

Conversely, under a high emission scenario (SSP585), its distribution pattern exhibits a vulnerable characteristic of “initial increase followed by decrease”. In the near term, while the area of highly suitable habitat shows a slight increase (67.99 × 10^4^ km^2^), it experiences a significant contraction by the end of the century, sharply declining to 46.13 × 10^4^ km^2^, with the overall suitable habitat retreating toward the core area of the Yangtze River basin and the southwestern ecological refugia. Meanwhile, the moderately suitable habitat expands eastward, forming a continuous belt extending from the southeastern hills to southeastern Tibet, reflecting potential shifts in species distribution and niche compression due to climate stress. The total suitable area decreases from 22.05% in the near term to 20.27% by the end of the century, representing a net reduction of 1.78 percentage points, which underscores the potential distribution risks posed to this species by severe climate change.

In summary, the future distribution of *C. paliurus* is not only constrained by rising temperatures but is also closely related to changes in precipitation patterns and increased seasonal drought. The contrast between the continuous expansion of suitable habitat under SSP126 and the later contraction under SSP585 highlights the critical importance of global carbon reduction efforts in maintaining the stability of distribution and ecological security for this rare species.

### 3.7. Analyzing Trends in Centroid Migration of C. paliurus’ Suitable Habitats Under Future Climate Scenarios

**Figure 8:**
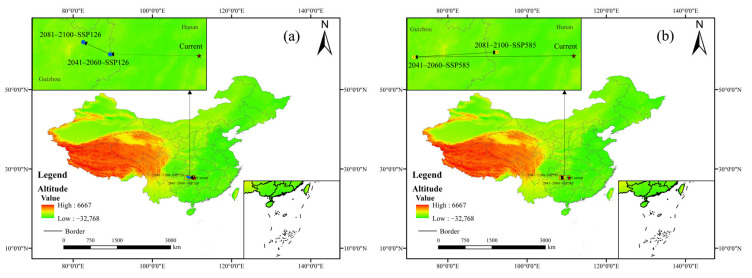
Centroid migration trajectories of the total suitable habitat for *C. paliurus* under future climate change. (**a**) 2050s–2090s, SSP126; (**b**) 2050s–2090s, SSP585.

To investigate the migration trends of *C. paliurus* distribution under future climate change scenarios, this study conducted a tracking analysis of the centroids of its suitable habitat distribution. The results indicate that under both climate scenarios, the centroids exhibit a trend of migrating towards higher elevation areas in the northwest (Figure 8, Table 6). This reflects the species’ adaptive strategy of retreating to regions with more favorable thermal and stable moisture conditions in response to climate warming. Under the SSP126 pathway, the centroid initially shifts westward by 132.57 km, followed by a continued northwest migration of 45.04 km, demonstrating a sustained and stable adaptive migration. In contrast, under the high-emission SSP585 scenario, the centroid displays a more erratic trajectory, initially migrating northwest over a distance of 238.58 km, and subsequently retreating northeast by 124.56 km. This suggests that under severe climate stress, the species distribution may exhibit instability, with its core survival area contracting towards ecological refuges such as the Yunnan–Guizhou Plateau. This difference clearly indicates that the intensity of climate change will directly affect the stability of species migration pathways, further underscoring the ecological importance of controlling greenhouse gas emissions to maintain stability in species distribution areas.

### 3.8. Correlation Between Chemical Composition and Environmental Variables

**Figure 9:**
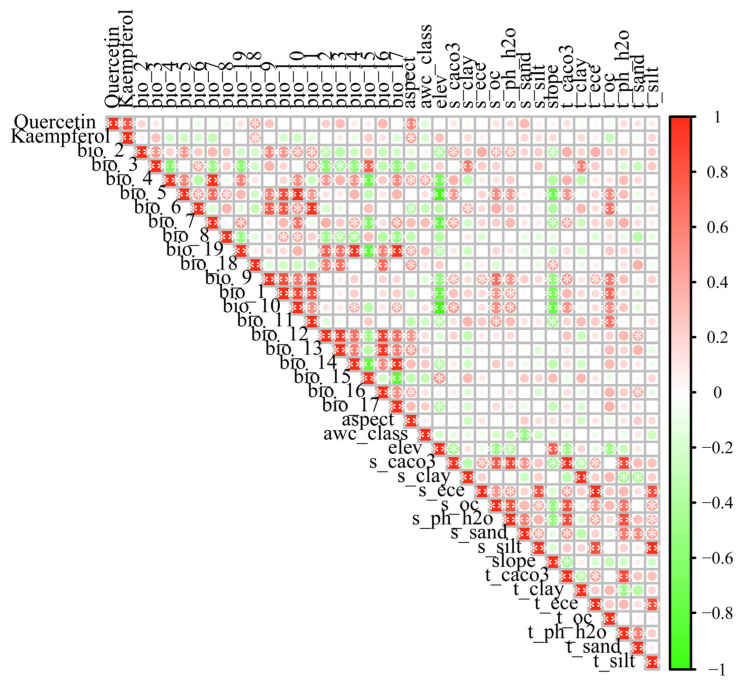
Results of correlation analysis between flavonoid components and environmental variables. The color intensity represents the strength of the correlation, with red indicating positive correlations and green indicating negative correlations. Darker shades correspond to stronger correlations. “*” indicates a *p*-value ≤ 0.05, “**” indicates a *p*-value ≤ 0.01.

Spearman correlation analysis indicated that the accumulation of quercetin and kaempferol in the leaves of *C. paliurus* is significantly positively correlated with two environmental factors: Precipitation of the Warmest Quarter (Bio18) and Aspect (*p* < 0.05) (Figure 9, Table 7). This statistical association suggests that water supply during the growing season and the light and thermal conditions regulated by slope orientation are key environmental indicators for predicting the spatial variation in flavonoid components in *C. paliurus*. These findings provide a direct basis for accurately delineating quality zones for medicinal materials based on environmental variables.

### 3.9. Integrated Quality Regions Evaluation

The validation results of the collaborative Kriging model indicate that the interpolation results are robust and reliable, making them suitable for subsequent spatial analyses. The mean prediction error for quercetin is close to zero (0.00077), with a root mean square error (RMSE) of 0.4227 and a normalized root mean square error (NRMSE) of 0.8852. For kaempferol, the mean prediction error is 0.2538, with an RMSE of 1.5055 and an NRMSE of 1.3490. The NRMSE values for both compounds are close to 1, suggesting that the model provides a reasonable estimation of prediction uncertainty. Although the error for kaempferol is slightly higher, likely due to its inherent variability in concentration, the overall accuracy still meets the requirements for spatial predictions.

**Figure 10:**
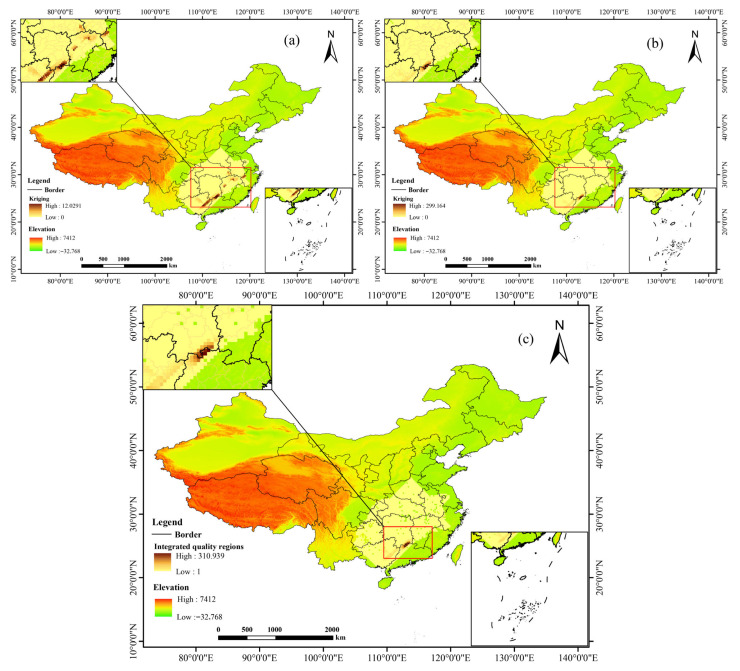
Integrated quality regions evaluation. (**a**) Co-kriging analysis of quercetin; (**b**) Co-kriging analysis of kaempferol; (**c**) Integrated quality regions of *C. paliurus*.

Based on the ecological suitability zones and the spatial distribution of flavonoid compounds (Figure 10a,b), integrated quality regions (Figure 10c) for *C. paliurus* were constructed. High quercetin concentration areas are primarily located in the junction of Guangdong, Hunan, and Guangxi provinces, as well as northern Jiangxi and western Zhejiang. In contrast, high kaempferol concentration areas are concentrated in the junction of Guangdong, Hunan, and Guangxi, as well as northwestern Zhejiang. These two regions exhibit both spatial overlap and complementarity. The integrated quality regions indicate that the core areas are continuously distributed in the junction of Guangdong, Hunan, and Guangxi, encompassing locations such as Yizhang, Linwu, Renhua, Rucheng, Lianshan Zhuang and Yao Autonomous County, and Jianghua Yao Autonomous County, extending to parts of Lechang, Jiahe, Shaoguan, Guilin, Chenzhou, and Ganzhou. Additionally, there are scattered high-quality patches in northern Jiangxi and other areas. This zoning result clearly delineates the potential high-quality resource areas for *C. paliurus*, providing a scientific basis for targeted cultivation and resource conservation.

## 4. Discussion

This study integrates ecological suitability and quality suitability models to elucidate the distributional shifts and driving mechanisms of *Cyclocarya paliurus* (Batal.) Iljinsk under future climate change scenarios. Geographic distribution models not only predict changes in species ranges but also play a crucial role in identifying key conservation areas and assessing the potential impacts of climate change on medicinal plant resources [35]. As demonstrated in the research on *Larix principis-rupprechtii*, incorporating multidimensional environmental factors such as climate, soil, and topography significantly enhances the accuracy of distribution predictions, thereby providing a scientific basis for regional ecological restoration efforts [36].

The habitat preferences of *C. paliurus* are highly consistent with the characteristics of subtropical native habitats documented in the literature [37]. Its current suitable areas, located in the middle and lower reaches of the Yangtze River and the southwestern region of China, overlap with the core distribution areas of the family Juglandaceae [38]. However, it differs in ecological niche characteristics from other species within the same family. For instance, members of the genus Juglans are more sensitive to drought stress [39], whereas *C. paliurus* relies on high humidity environments, with Bio17 contributing 34% to its habitat suitability. This correlation aligns with existing studies indicating that suitable areas for *C. paliurus* are characterized by annual precipitation exceeding 1200 mm [22]. This trait renders *C. paliurus* particularly sensitive to changes in precipitation patterns, exhibiting a trend of migration towards higher altitudes and latitudes under the SSP scenarios [22].

The predicted northwest migration of *C. paliurus* towards the Yunnan–Guizhou Plateau not only reflects its ecological niche conservatism but also reveals the complex survival challenges that this species may face under future climate pressures. Historical studies indicate that species in the East Asian monsoon region often mitigate climate stress through topographical heterogeneity [40]. The complex mountainous terrain of the Yunnan–Guizhou Plateau is expected to serve as a climate refuge for *C. paliurus* in the future, a conclusion that aligns with its residual distribution characteristics during glacial refuge periods [22]. Models also predict that the Pearl River Basin, including southeastern Guizhou and southwestern Hunan, will form new suitable habitats, spatially connecting with traditional distribution areas such as Jiangxi and Zhejiang. However, the prevalent risk of soil acidification in future expansion areas, such as the karst region of southern Guizhou, may limit the effectiveness of cultivation [22], necessitating appropriate soil improvement measures. In addition to climate change and soil acidification, *C. paliurus* may also be threatened by various environmental factors within its suitable habitats. Existing research indicates that habitat fragmentation caused by land-use changes could directly compress the effective living space of the species and hinder gene flow between populations [41]. Air pollution, such as increased ozone concentrations, may disrupt photosynthetic efficiency by inducing oxidative stress responses in plants [42]. Furthermore, climate warming could lead to an increase in the frequency and intensity of pests and diseases [43], while the invasion of non-native species may further destabilize native ecosystems [44]. Should these non-climatic stressors interact with climate change, they could accelerate the decline of local populations.

The ecological adaptability of *C. paliurus* exhibits distinct specificity among medicinal plants. In comparison to salt-tolerant medicinal species such as *Apocynum venetum* [45], *C. paliurus* demonstrates significantly lower salt tolerance [46]. This characteristic is particularly critical under the SSP585 scenario, as prolonged dry seasons may exacerbate the risk of soil salinization [47]. Similarly to its congener *Juglans regia*, *C. paliurus* prefers moist habitats; however, *Juglans regia* displays a broader range of seasonal temperature adaptability [48]. Notably, *C. paliurus* exhibits unique ecological niche characteristics when compared to other medicinal plants. In contrast to deep-rooted medicinal species such as *Astragalus membranaceus* [49], *C. paliurus* is more sensitive to water stress, which may limit its distribution in seasonally arid regions. Additionally, compared to broadly adaptable medicinal species like *Lycium barbarum* [50,51], *C. paliurus* has a noticeably narrower ecological niche width. These comparisons suggest that *C. paliurus* may face greater challenges in responding to extreme events induced by future climate change. Based on these findings, future breeding strategies should focus on the exploration of genetic resources for stress resistance, particularly key enzymes in the triterpene biosynthetic pathway (OSCs) [52,53], while also leveraging identified stress-resistance gene resources from other medicinal plants [54,55] to enhance *C. paliurus*’s adaptability to complex environmental stresses.

The flavonoid compounds abundant in the leaves of *C. paliurus* constitute a significant basis for its medicinal value, with quercetin and kaempferol glycosides (such as isoquercitrin and kaempferol-3-O-glucoside) identified as the primary active components [56,57]. Quercetin, a polyhydroxy flavonoid, exhibits a variety of pharmacological effects including antioxidant, anti-inflammatory, antiviral, antitumor, and hypoglycemic activities [58,59]. Similarly, kaempferol demonstrates extensive biological activity, including anti-inflammatory, anticancer, hepatoprotective effects, and the ability to combat obesity and diabetes, as well as to inhibit vascular endothelial inflammation and protect neurological and cardiac functions [60]. These notable pharmacological activities provide a scientific basis for the use of *C. paliurus* in traditional medicine. The accumulation of these compounds was found to be jointly regulated by genetic background, ecological factors, and practical operational methods [56,61]. Correlation analysis revealed a significant statistical association between the contents of quercetin and kaempferol and the precipitation of the warmest quarter (Bio18) as well as aspect. We cautiously suggest that precipitation of the warmest quarter may indirectly influence secondary metabolism by alleviating heat stress [62], while south-facing aspect are generally associated with better light and temperature conditions [63]. It is important to emphasize that this association indicates a spatial covariation between environmental factors and compound accumulation, and the precise causal mechanisms require further validation through controlled experiments.

The concept of “integrated quality regions” proposed in this study refers to areas that simultaneously meet the ecological suitability for medicinal materials and the spatial requirements for the enrichment of active components [64]. This concept aligns with the notion of “genuine producing areas” in the research of authentic medicinal materials [65], which involves identifying regions that can ensure resource sustainability while optimizing quality through spatial analysis methods [66]. This zoning can be translated into three functional areas: priority protection zones (where strict habitat protection is implemented and commercial collection of wild resources is restricted) [67], standardized cultivation core areas (where standardized planting techniques are promoted) [68], and ecological and economic synergistic development zones (where efficient ecological models, such as forest-medicine intercropping, are explored) [69]. This zoning framework enhances quality through optimized cultivation practices and provides a scientific basis for resource conservation and sustainable utilization.

This study has several limitations that should be addressed in future research. First, the sample size for chemical composition analysis is limited (n = 26). Although this sample encompasses the main distribution areas, it restricts the predictive accuracy and generalizability on a national scale. Sample size limitations are often considered a significant constraint [70], potentially affecting the robustness of the model results [71]. Second, species distribution models (SDMs) inherently contain uncertainties [72]. These include spatial biases in species occurrence records and variability in future climate scenarios (Shared Socioeconomic Pathways, SSPs) [73,74]. It has been demonstrated that spatial bias in training data can diminish the predictive performance of SDMs [70], and this spatial distribution characteristic can sometimes be more critical than the actual sample size [75]. Additionally, uncertainties in future scenarios will also propagate into the predictions [76]. Furthermore, the interaction mechanisms between environmental factors and the accumulation of chemical components, as well as the biosynthetic pathways of key pharmacologically active compounds, require further elucidation. The complexity of this field necessitates the integration of multi-omics approaches, such as transcriptomics and metabolomics, for more in-depth investigation [77,78]. Based on the aforementioned limitations, future research could focus on the following aspects: First, systematically expanding the spatial sampling scale of chemical components in *C. paliurus*. Existing studies have shown that sampling density directly influences the ability to capture environmental heterogeneity [79]; increasing the sample size will effectively enhance model performance and the generalizability of predictions. Second, integrating multiple environmental factors. For instance, incorporating anthropogenic disturbances and other significant factors into niche modeling may improve predictive accuracy [80]. Third, incorporating population genetics methods. This approach has been widely applied to analyze the genetic basis of population structure and adaptive differentiation in species [81]. Future studies could investigate the genetic variation among *C. paliurus* populations across different quality gradients to further reveal the mechanisms underlying their ecological adaptability, thereby providing a theoretical basis for the conservation of genetic resources and targeted quality improvement.

*C. paliurus*, a glacial relict tree species from the Quaternary period, is an endemic and rare plant in China, often referred to as the “panda of the plant kingdom” [82]. Its medicinal value is significant, and it has been recognized as the third major milestone species in the medical field, following aspirin and paclitaxel [83]. Although this species is primarily distributed across several provinces in southern China, it currently exists mainly in fragmented natural forests with limited population sizes [84]. In recent years, habitat fragmentation and overexploitation have led to a rapid decline in wild resources, highlighting the urgent need for systematic conservation strategies. Conservation measures should encompass three key aspects: (1) implementing habitat protection and strictly controlling the collection of wild resources; (2) promoting the industrialization of artificial cultivation, with a focus on establishing planting bases in regions characterized by high annual precipitation, suitable average temperatures, and south-facing slopes; and (3) strengthening the foundation of research systems, particularly focusing on the collection and preservation of genetic resources, the functional analysis of key genes [85], and cultivation physiology studies related to light quality regulation [86]. It is recommended that forestry departments establish a collaborative regulatory mechanism involving multiple sectors. This can be achieved through the establishment of genetic resource banks, the promotion of standardized planting techniques, and the targeted cultivation of medicinal components, thereby facilitating sustainable development in both ecological conservation and resource utilization.

## 5. Conclusions

This study systematically evaluates the ecological suitability and spatial distribution of medicinal components of *C. paliurus* by integrating species distribution models with geostatistical methods. The main conclusions and management recommendations are as follows: (1) The distribution of *C. paliurus* is primarily regulated by precipitation and temperature factors, with the precipitation of the driest quarter, annual precipitation, and minimum temperature of the coldest month identified as key limiting factors. It is recommended that areas with stable hydrological and thermal conditions be prioritized in the planning of conservation and cultivation zones to enhance the climate resilience of populations. (2) The current suitable area for *C. paliurus* is approximately 1.91 million square kilometers, concentrated in provinces such as Jiangxi, Zhejiang, and Hunan. In the future, the centroid of suitable areas is expected to shift northwestward, with the Yunnan–Guizhou Plateau potentially serving as an important climate refuge. It is advised to expand the conservation areas from the existing core regions to include ecological corridors along the Nanling–Wuyi Mountains and the migration routes. (3) The delineation of integrated quality regions indicates that the border area of Guangdong, Hunan, and Guangxi (centered around Yizhang County) represents a high-quality resource potential zone. This area is suitable for standardized cultivation and the production of high-quality raw materials, promoting a precise cultivation model characterized by “optimal land, optimal production, and optimal quality”. (4) In response to the decline of wild resources, it is recommended to establish a triadic strategy of “in situ conservation–nearby cultivation–germplasm innovation”. This includes establishing protection zones in core areas with restricted collection, creating ecological cultivation demonstration zones along migration corridors, and enhancing germplasm resource banks along with the exploration of resilient, high-quality genes. It should be noted that this study is limited by the sample size of chemical analyses, spatial sampling biases, and uncertainties in climate scenarios. Nonetheless, the proposed ecological-quality dual-dimensional zoning framework can provide a scientific basis for the conservation of *C. paliurus* resources, prioritization of zoning, and industrial layout. It is recommended that forestry, agriculture, and pharmaceutical regulatory departments collaborate to establish a long-term mechanism for resource monitoring, germplasm management, and standardized production to achieve a balance between conservation and sustainable use.

## Figures and Tables

**Table 1:** All environmental variables.

Variable	Name	Unit
Bio1	Annual mean temperature	°C
Bio2	Mean diurnal temperature range	°C
Bio3	Isothermality	/
Bio4	Temperature seasonality	/
Bio5	Maximum temperature of the warmest month	°C
Bio6	Minimum temperature of the coldest month	°C
Bio7	Mean temperature of the wettest quarter	°C
Bio8	Mean temperature of the wettest quarter	°C
Bio9	Mean temperature of the driest quarter	°C
Bio10	Mean temperature of the warmest quarter	°C
Bio11	Mean temperature of the coldest quarter	°C
Bio12	Annual precipitation	mm
Bio13	Precipitation of the wettest month	mm
Bio14	Precipitation of the driest month	mm
Bio15	Precipitation seasonality	/
Bio16	Precipitation of the wettest quarter	mm
Bio17	Precipitation of the driest quarter	mm
Bio18	Precipitation of the warmest quarter	mm
Bio19	Precipitation of the coldest quarter	mm
Awc_class	Soil available water content	%
Slope	Slope	◦
Elev	Elevation	m
Aspect	Aspect	/
T_ph_h2o	Topsoil pH	−log(H^+^)
S_ph_h2o	Subsoil pH	−log(H^+^)
T_oc	Topsoil organic carbon content	%weight
S_oc	Subsoil organic carbon content	%weight
T_clay	Topsoil clay content	%weight
S_clay	Subsoil clay content	%weight
T_sand	Topsoil sand content	%weight
S_sand	Subsoil sand content	%weight
T_silt	Topsoil silt content	%weight
S_silt	Subsoil silt content	%weight
T_ece	Topsoil electrical conductivity	ds/m
S_ece	Subsoil electrical conductivity	ds/m
T_caco3	Topsoil carbonate or lime content	%weight
S_caco3	Subsoil carbonate or lime content	%weight

**Table 2:** Environmental predictors for MaxEnt model.

Variables	Name	Unit
Bio2	Mean diurnal range (Mean of monthly (max temp-min temp))	°C
Bio4	Temperature seasonality	/
Bio5	Maximum temperature of warmest month	°C
Bio6	Minimum temperature of coldest month	°C
Bio8	Mean temperature of wettest quarter	°C
Bio12	Annual precipitation	mm
Bio15	Precipitation seasonality	/
Bio17	Precipitation of driest quarter	mm
Bio18	Precipitation of warmest quarter	mm
S_oc	Substratesoil organic carbon	% weight
S_ph_h2o	Substratesoil pH	−log(H^+^)
T_oc	Topsoil organic carbon	% weight
T_silt	Topsoil silt content	%
T_sand	Topsoil sand content	%
T_clay	Topsoil clay content	% weight
Aspect	Aspect	/
Slope	Slope	◦

**Table 3:** Environmental variables contributions.

Variables	Name	Percent Contribution (%)
Bio2	Mean diurnal range (Mean of monthly (max temp-min temp))	1.4
Bio4	Temperature seasonality	5.4
Bio5	Maximum temperature of warmest month	0.6
Bio6	Minimum temperature of coldest month	11.3
Bio8	Mean temperature of wettest quarter	3.6
Bio12	Annual precipitation	32.0
Bio15	Precipitation seasonality	0.4
Bio17	Precipitation of driest quarter	34.0
Bio18	Precipitation of warmest quarter	0.4
S_oc	Substratesoil organic carbon	0.2
S_ph_h2o	Substratesoil pH	2.2
T_oc	Topsoil organic carbon	0.6
T_silt	Topsoil silt content	0.5
T_sand	Topsoil sand content	2.4
T_clay	Topsoil clay content	0.8
Aspect	Aspect	1.3
Slope	Slope	3.0

**Table 4:** The suitable range for the dominant environmental variables.

Variable	Suitable Range	Adaptive Threshold
Bio17	57.7~589.6 mm	563.2 mm
Bio12	1078.4~2172.6 mm	1280.5 mm
Bio6	−1.9~6.8 °C	2.1 °C
Bio4	594.3~879.4	807.3
Bio2	6.9~9.2 °C	8.3 °C

**Table 5:** Distribution patterns of *C. paliurus* under varying climate projections and temporal scales.

Periods	Climate Scenarios	Highly Suitable (×10^4^ km^2^)	Moderately Suitable (×10^4^ km^2^)	Generally Suitable (×10^4^ km^2^)	Total Suitable Area (×10^4^ km^2^)
2050s	SSP126	61.21	62.58	82.88	206.67
	SSP585	67.99	60.47	83.49	211.94
2090s	SSP126	74.57	60.38	88.40	223.35
	SSP585	46.13	69.15	79.50	194.77

**Table 6:** Changes in the centroid of *C. paliurus* under different climatic scenarios over periods.

Climate Scenarios	Periods	Longitude (°E)	Latitude (°N)	Migration Distance (km)
	Present	110.65	27.79	
SSP126	2050s	109.31	27.81	132.57
SSP126	2090s	108.90	28.00	45.04 (2050s to 2090s)
SSP585	2050s	108.23	27.77	238.58
SSP585	2090s	109.49	27.84	124.56 (2050s to 2090s)

**Table 7:** Correlation coefficients between flavonoid chemical constituents and environmental variables.

Variable	Quercetin	Kaempferol
Precipitation of the warmest quarter (Bio18)	0.432 *	0.390 *
Aspect	0.584 **	0.458 *

**. correlation is significant at the 0.01 level (two-tailed); *. correlation is significant at the 0.05 level (two-tailed).

## Data Availability

Data can be made available on reasonable request.

## Supplementary Materials

The following supporting information can be downloaded at: https://www.mdpi.com/article/10.3390/biology14121639/s1, Table S1: Composition data of active components in Cyclocarya paliurus from different geographical origins.
