# Supplementary material for: Establishment of Integrated Quality Regions for the Rare Medicine Food Homology Plant Cyclocarya paliurus (Batal.) Iljinsk in China
Source: Biology (Basel). 2025 Nov 21;14(12):1639. doi: 10.3390/biology14121639 (PMC12729511; doi:10.3390/biology14121639)
Supplement: Supplementary file 1 [file biology-14-01639-s001.zip › biology-3977481-supplementary.pdf]

**Supplementary Table S1:** Composition data of active components in *Cyclocarya paliurus* from different geographical origins.

| Sample | Geographic Origin | Longitude (°E) | Latitude (°N) | Quercetin Content (mg/g) | Kaempferol Content (mg/g) | Collection Time |
|---|---|---|---|---|---|---|
| S1 | Jianhe County, Qiandongnan City, Guizhou Province, China | 108.44752 | 26.73405 | 0.0756 | 0.0756 | 2016 |
| S2 | Wuhu City, Anhui Province | 118.373117 | 31.301906 | 0.1562 | 0.106 | 2020 |
| S3 | Huangshan District, Huangshan City, Anhui Province | 118.146689 | 30.085511 | 0.1686 | 0.1084 | 2020 |
| S4 | Anji County, Huzhou City, Zhejiang Province | 119.68656 | 30.64442 | 1.218 | 1.833 | 2008 |
| S5 | Jianghua County, Yongzhou City, Hunan Province | 111.58545 | 25.19135 | 2.696 | 4.882 | 2008 |
| S6 | Kunming City, Yunnan Province | 102.83944 | 24.88627 | 1.131 | 1.361 | 2008 |
| S7 | Qingliangfeng, Jixi County, Xuancheng City, Anhui Province | 118.85815 | 30.14559 | 1.189 | 2.344 | 2008 |
| S8 | Lushan District, Jiujiang City, Jiangxi Province | 115.98446 | 29.5703 | 1.389 | 2.561 | 2008 |
| S9 | Longlin County, Baise City, Guangxi Province | 105.35046 | 24.77681 | 1.6912 | 2.7263 | 2021 |
| S10 | Xiushui County, Jiujiang City, Jiangxi Province | 114.55357 | 29.03118 | 0.444 | 0.148 | 2018 |
| S11 | Danjiang Town, Leishan County, Guizhou Province | 108.08863 | 26.3817 | 0.2443 | 0.2124 | 2015 |
| S12 | Datang Town, Leishan County, Guizhou Province | 108.07433 | 26.32541 | 0.0858 | 0.2183 | 2015 |
| S13 | Pingyang Township, Rongjiang County, Guizhou Province | 108.35555 | 26.3017 | 0.1793 | 0.2145 | 2015 |
| S14 | Yongle Town, Leishan County, Guizhou Province | 108.22757 | 26.23191 | 0.1969 | 0.1987 | 2015 |
| S15 | Resources County, Guilin City, Guangxi Province | 110.65844 | 26.04857 | 0.2138 | 0.0782 | 2016 |
| S16 | Sangzhi County, Zhangjiajie City, Hunan Province | 110.17042 | 29.40592 | 0.2778 | 0.3189 | 2016 |
| S17 | Tonggu County, Yichun City, Jiangxi Province | 114.36099 | 28.51966 | 0.2471 | 0.1528 | 2016 |
| S18 | Angying Village, Jianhe County, Guizhou Province | 108.35184 | 26.37291 | 0.12703 | 0.15552 | 2015 |
| S19 | Suining County, Shaoyang City, Hunan Province | 110.16242 | 26.58842 | 0.05395 | 0.06185 | 2018 |
| S20 | Liuyang City, Hunan Province | 113.6494 | 28.16983 | 0.0518 | 0.0451 | 2018 |
| S21 | Enshi City, Hubei Province | 109.48655 | 30.30104 | 0.0708 | 0.0364 | 2018 |
| S22 | Xiushui County, Jiujiang City, Jiangxi Province | 114.55357 | 29.03118 | 0.1961 | 0.7939 | 2018 |
| S23 | Hefeng County, Enshi City, Hubei Province | 110.04042 | 29.89612 | 0.1165 | 0.1212 | 2018 |
| S24 | Wugang City, Shaoyang City, Hunan Province | 110.63841 | 26.7329 | 0.06693 | 0.05567 | 2018 |
| S25 | Lueyang County, Hanzhong City, Shaanxi Province | 106.16354 | 33.33319 | 0.16485 | 0.17995 | 2018 |
| S26 | Jiuhua Mountain, Qingyang County, Chizhou City, Anhui Province | 117.83051 | 30.48489 | 0.0975 | 0.0874 | 2020 |
